# Pathological Analysis of Ocular Lesions in a Murine Model of Sjögren’s Syndrome

**DOI:** 10.3390/ijms18061209

**Published:** 2017-06-06

**Authors:** Aya Ushio, Rieko Arakaki, Hiroshi Eguchi, Fumika Hotta, Akiko Yamada, Yasusei Kudo, Naozumi Ishimaru

**Affiliations:** 1 Departmant of Oral Molecular Pathology, Tokushima University Graduate School of Biomedical Sciences, Tokushima 770-8504, Japan; c301451013@tokushima-u.ac.jp (A.U.); arakaki.r@tokushima-u.ac.jp (R.A.); aki.yamada@tokushima-u.ac.jp (A.Y.); yasusei@tokushima-u.ac.jp (Y.K.); 2 Department of Ophtalmology, Sakai Hospital Kindai University, Osaka 590-0132, Japan; hiroegu0113@gmail.com (H.E.); uri0428@yahoo.co.jp (F.H.)

**Keywords:** Sjögren’s syndrome, keratoconjunctivitis, dry eye, tears, lacrimal glands, cytokines, chemokines

## Abstract

Sjögren’s syndrome (SS) is a systemic autoimmune disease characterized by severe inflammation of exocrine glands such as the salivary and lacrimal glands. When it affects the lacrimal glands, many patients experience keratoconjunctivitis due to severely dry eyes. This study investigated the pathological and immunological characteristics of ocular lesions in a mouse model of SS. Corneal epithelial injury and hyperplasia were confirmed pathologically. The number of conjunctival mucin-producing goblet cells was significantly decreased in the SS model mice compared with control mice. Expression levels of transforming growth factor (TGF)-β, interleukin (IL)-6, tumor necrosis factor (TNF)-α, and C-X-C motif chemokine (CXCL) 12 were significantly higher in the corneal epithelium of the SS model mice than in control mice. Inflammatory lesions were observed in the Harderian, intraorbital, and extraorbital lacrimal glands in the SS model mice, suggesting that the ocular glands were targeted by an autoimmune response. The lacrimal glands of the SS model mice were infiltrated by cluster of differentiation (CD)4^+^ T cells. Real-time reverse transcription-polymerase chain reaction (RT-PCR) revealed significantly increased mRNA expression of TNF-α, TGF-β, CXCL9, and lysozyme in the extraorbital lacrimal glands of the SS model mice compared with control mice. These results add to the understanding of the complex pathogenesis of SS and may facilitate development of new therapeutic strategies.

## 1. Introduction

Sjögren’s syndrome (SS) is an autoimmune disease affecting the exocrine glands, including the lacrimal and salivary glands, causing dry eyes and mouth symptoms such as severe keratoconjunctivitis and oral disorders [1]. Many SS patients suffer from burning, itchiness, eye pain, or tired eyes [2,3]. Because the pathogenesis of SS is complex, the main therapy for SS has been treatments of the symptoms [4].

The pathogenesis and molecular mechanisms of SS have been investigated in various animal models of SS [5], including a thymectomized neonatal NFS/*sublingual gland* (*sld*) mutant mouse model, which we developed to study SS and the therapeutic effects of several reagents on its autoimmune lesions [6,7,8,9,10]. The detailed ocular lesions in the eyeball, cornea, iris, and lacrimal gland lesions in the mouse models are not fully understood due to the complicated anatomy and small structures.

In this study, lesions of the cornea and lacrimal glands produced in the mouse model were evaluated pathologically and ocular lesions were evaluated immunologically to broaden our understanding of the pathogenesis of SS autoimmunity.

## 2. Results

### 2.1. Dry Eye in Sjögren’s Syndrome (SS) Model Mice

#### 2.1.1. Tear Secretion

**Figure 1:**
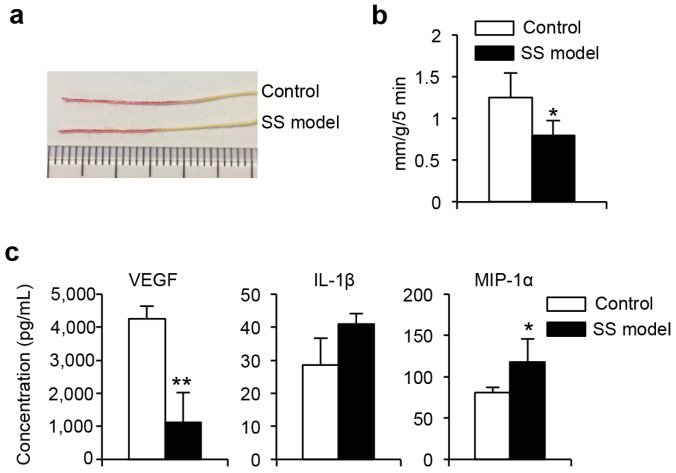
Dry eye in the Sjögren’s syndrome (SS) model mice. (**a**) Tear volume was determined by measuring the length of the phenol-red threads remaining in contact with the eye at 5-min intervals after intraperitoneal injection of pilocarpine in anesthetized mice; (**b**) Tear volumes were reported as tear volume/body weight at 10 min. Data are means ± standard deviation (SD) of eight mice for each group; and (**c**) Twenty cytokines, chemokines, and growth factors were quantified in tears using the Luminex assay. Vascular endothelial growth factor (VEGF), interleukin (IL)-1β, and macrophage inflammatory protein (MIP)-1α concentrations are means ± SD of five mice for each group. * *p* < 0.05, ** *p* < 0.005.

Tear secretion volume was determined in anesthetized mice by measuring the length of the phenol-red thread left in contact with the eye at 5 and 10 min after intraperitoneal injection of pilocarpine. Tear secretion volume of these SS model mice at 10 weeks of age was significantly greater than that of age-matched control mice (Figure 1a,b). The dry eyes of these SS model mice were confirmed as previously described [11].

#### 2.1.2. Cytokines, Chemokines, and Growth Factors in Tears

The levels of cytokines, chemokines, and growth factors present in tears, including granulocyte-macrophage colony-stimulating factor (GM-CSF), interferon (IFN)-γ, interleukin (IL)-1α, IL-1β, IL-2, IL-4, IL-5, IL-6, IL-10, IL-12, IL-13, IL-17, tumor necrosis factor (TNF)-α, C-X-C motif ligand (CXCL)10, CXCL1, monocyte chemotactic protein-1 (MCP-1), CXCL9, macrophage inflammatory protein (MIP)-1α, basic fibroblast growth factor (FGF), and vascular endothelial growth factor (VEGF), were determined using a Luminex assay. VEGF was significantly lower in the SS model mice than in the control mice (Figure 1c), and MIP-1α was significantly higher compared to the control mice. There were no differences in the other factors, such as IL-1β, in the SS model and control mice (Figure 1c).

#### 2.1.3. Corneal Epithelium

**Figure 2:**
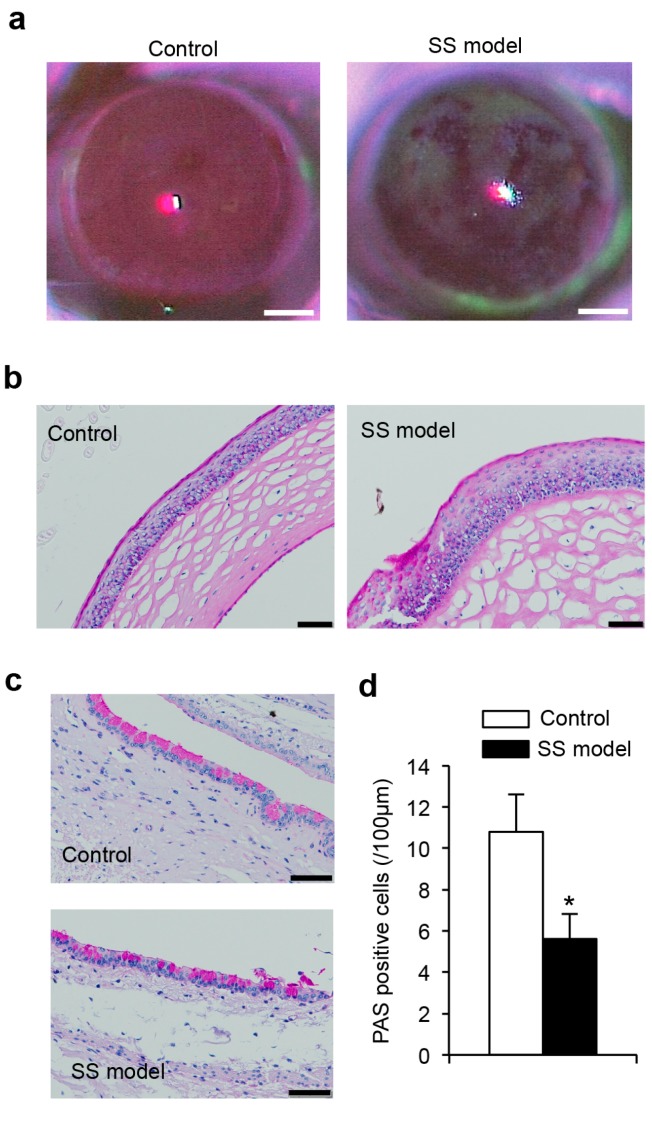
Keratoconjunctivitis of the SS model mice. (**a**) One to two μL of fluorescein solution was applied to the cornea by micropipette. The residual liquids around the eye were washed with saline and gently wiped off using filter paper. Corneas were examined for epithelial disorders using a slit-lamp microscope with a blue filter. The results are representative of five mice in each group. Scale bar: 1 mm; (**b**) Histological findings of corneal epithelia. Sections of corneal tissues of the control and the SS model mice were stained with (periodic acid-Schiff) PAS. Photos are representative of five mice for each group. Scale bar: 50 μm; (**c**) PAS^+^ mucin-producing goblet cells in conjunctival tissues of control and SS model mice are shown. Photos are representative of five mice in each group. Scale bar: 50 μm; and (**d**) The number of PAS^+^ mucin-producing goblet cells in conjunctival tissues of control and SS model mice is shown. The data are means ± SD of five mice in each group. * *p* < 0.05.

Mice were assessed for corneal epithelial disorders by fluorescein staining. The corneal epithelium of SS model mice showed sparse or dense superficial punctate keratitis similar to that of SS patients. The corneal epithelium of control mice was not detectably stained (Figure 2a). This finding confirmed corneal injury resulting from dry eye in these SS model mice.

#### 2.1.4. Histology of Corneal Epithelium

The surface of the corneal epithelium of the SS model mice was irregular, whereas that of control mice was smooth (Figure 2b). In addition, enhanced keratinization of the epithelium and hyperplasia of epithelial cells were observed in the SS model mice (Figure 2b).

#### 2.1.5. Conjunctival Goblet Cells

Mucin secretion was assayed by the number of periodic acid-Schiff (PAS)-stained conjunctival goblet cells, which was significantly decreased in the SS model mice compared with control mice (Figure 2c,d).

#### 2.1.6. Gene Expression in Corneal Epithelium

**Figure 3:**
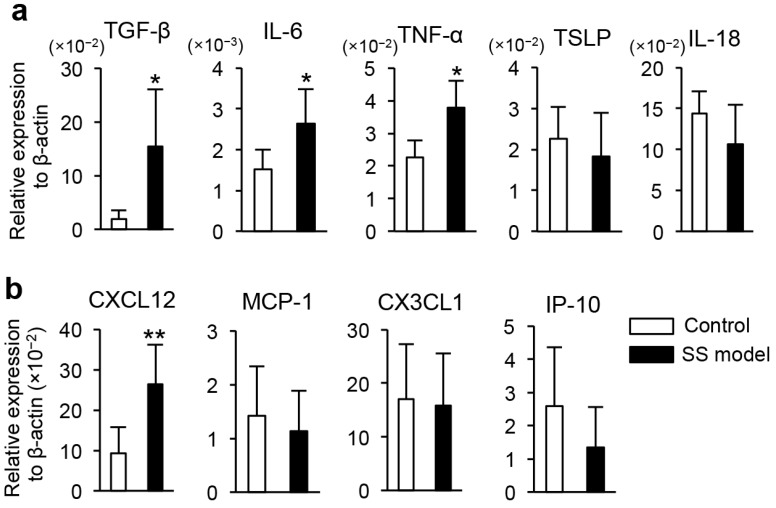
Gene expression in corneal tissues of the SS model mice. (**a**) mRNA expression of cytokines in corneal tissues from the SS model and control mice was determined by real-time reverse transcription-polymerase chain reaction (RT-PCR); and (**b**) mRNA expression of chemokines in the corneal tissues was determined by real-time RT-PCR. Data are means ± SD of six mice in each group. * *p* < 0.05, ** *p* < 0.01.

Cytokine and chemokine mRNA expression in the corneal epithelium of mouse eyeballs was assayed using a real-time reverse transcription-polymerase chain reaction (RT-PCR). Expression levels of *TGF-β*, *IL-6*, and *TNF-α* mRNA were significantly higher in the SS model mice compared to the control mice (Figure 3a). There were no differences in the mRNA expression of other cytokines, such as *thymic stromal lymphopoietin* (*TSLP*) or *IL-18* (Figure 3a). The expression of chemokine *CXCL12* mRNA was significantly increased in the SS model mice compared with control mice (Figure 3b). There were no differences in the expression of the other cytokine mRNAs, such as *MCP-1*, *CX3CL1*, or *CXCL10* in the SS model and control mice (Figure 3b).

### 2.2. Inflammatory Lesions of Lacrimal Glands in SS Model Mice

#### 2.2.1. Pathology of Lacrimal Glands

**Figure 4:**
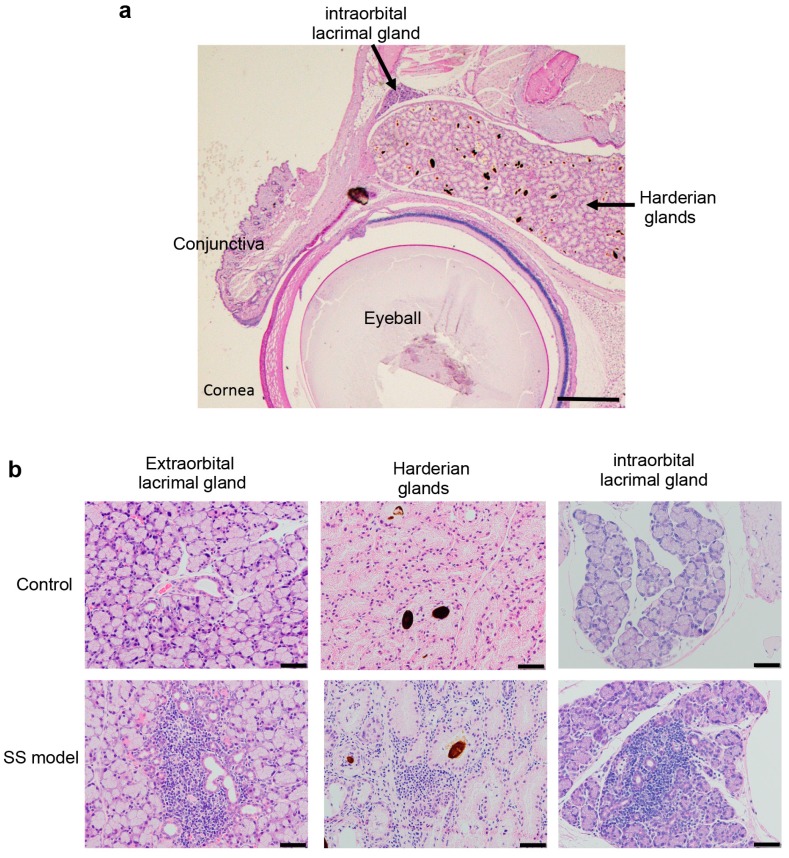
Intraorbital tissues of SS model mice. (**a**) Histological structure of the intraorbital tissues of control mice. Scale bar: 1 mm; and (**b**) Inflammatory lesions in the extraorbital lacrimal glands, Harderian glands, and intraorbital lacrimal glands are shown. Photos are representative of eight mice in each group. Scale bar: 50 μm.

Figure 4a shows a Harderian gland present in the intraorbital tissue of a control mouse [12], with brown pigment in the acinar lumens, and an adjacent intraorbital lacrimal gland. The extraorbital lacrimal glands are the primary tear-secreting tissue. Extensive lymphocyte infiltration around expanded ducts, and degeneration or destruction of the acinar cells were observed in the extraorbital lacrimal glands of SS model mice (Figure 4b). Inflammatory lesions were also observed in the Harderian glands and intraorbital lacrimal glands of the SS model mice. No such lesions were seen in control mice (Figure 4b). The pathological findings demonstrate that Harderian glands, and both intraorbital and extraorbital lacrimal glands were targets of the autoimmune response in the SS model mice.

#### 2.2.2. Immunological Analysis of Extraorbital Lacrimal Glands in SS Model

**Figure 5:**
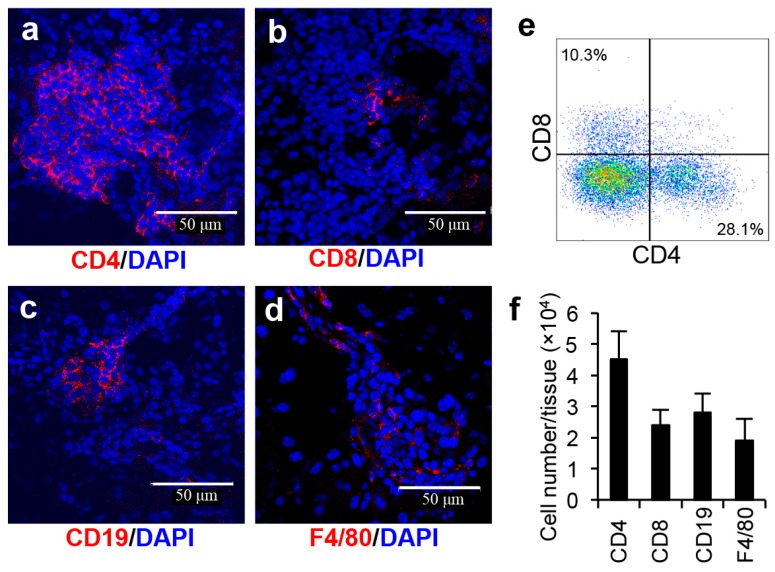
Immune cell infiltration of target organs of the SS model mice. CD4, CD8, CD19, and F4/80 expressing immune cells were observed by confocal microscopy. (**a**) CD4^+^ T cells, (**b**) CD8^+^ T cells, (**c**) CD19^+^ B cells, and (**d**) F4/80^+^ macrophages in the lacrimal glands of the SS model mice were detected by Alexa-546-conjugated antibodies. Nuclei were stained with 4′,6-diamdino-2-phenylindole dihydrochloride (DAPI). The results are representative of five mice. Scale bar: 50 μm; (**e**) Flow cytometry analysis using lacrimal gland tissues from the SS model mice. CD4 and CD8 T cell subsets ware shown, and the result is representative of 5 mice; and (**f**) Cell numbers of CD4^+^, CD8^+^ T cells, CD19^+^ B cells, and F4/8^+^ macrophages were calculated by flow cytometery analysis. Data are means ± SD of 5 mice.

Immunofluorescence assays characterized the immune cell population in the inflammatory lesions of the SS model mice. The predominant immune cell subset in the lacrimal glands of the SS model mice consisted of CD4^+^ T cells (Figure 5a). A few CD8^+^ T cells, CD19^+^ B cells, and F4/80^+^ macrophages were detected in the lesions (Figure 5b–d). To quantify the proportion and cell numbers of immune cell subsets precisely, we performed flow cytometry analyses using mononuclear cells in lacrimal glands from the SS model. The proportion of CD4^+^ T cells was higher than CD8^+^ T cells (Figure 5e). Cell numbers of CD4^+^ T cells within the extraorbital lacrimal gland tissue in the SS model mice was highest among the immune cell subsets (Figure 5f). The findings were consistent with many previous reports about the SS models and patients [1,4,5].

#### 2.2.3. Change in Gene Expression in Extraorbital Lacrimal Glands in the SS Model Mice

**Figure 6:**
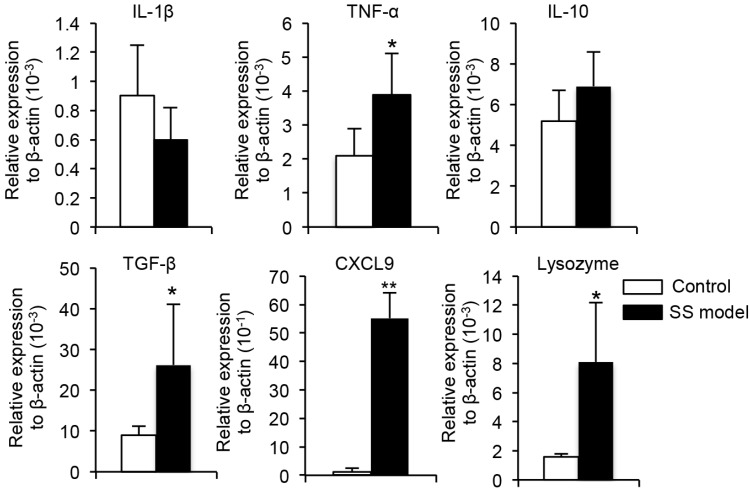
Gene expressions were assayed by RT-PCR. Expression of *IL-1β*, *TNF-α*, *IL-10*, *TGF-β*, *CXCL9*, and *lysozyme* mRNA is shown. Data are average RNA levels ± SD of five mice in each group. * *p* < 0.05, ** *p* < 0.005.

To investigate SS pathogenesis, mRNA expression of various cytokines, chemokines, and exocrine gland-related factors were assayed in the extraorbital lacrimal glands using RT-PCR. *TNF-α* and *TGF-β* cytokine mRNA expression and *CXCL9* chemokine mRNA expression were significantly upregulated in the SS model mice compared with control mice (Figure 6). *Lysozyme* mRNA expression was also significantly increased in the SS model mice compared with control mice (Figure 6).

## 3. Discussion

Pathological analysis in the present study using a murine model of SS revealed the injury of the corneal epithelium and inflammatory lesions in the Harderian and intraorbital lacrimal glands in addition to the extraorbital lacrimal glands. In addition, the increased expression of inflammatory cytokines and chemokines in the corneal epithelium and lacrimal glands was related to the pathogenesis of SS. Although it has been still unclear why the exocrine glands such as the lacrimal gland, are targeted in the SS model, as well as in human SS patients, investigation using an accurate model resembling SS may be useful for understanding the cellular and molecular mechanisms of the complicated pathogenesis of SS. Among the various mouse models of SS, autoimmune lesions in the model in this study were observed to occur at a high incidence, and analysis of autoimmune lesions based on immune dysfunction can lead to clinical applications for human autoimmunity.

The pathogenesis of SS is complex and multifactorial, involving immune-system dysfunction with attacks on self-cells, tissues, and organs [1]. The target organs are exocrine glands, including the salivary and lacrimal glands. The primary clinical symptoms are those of sicca syndrome, including dry eyes (xerophthalmia) and mouth (xerostomia) [4]. Extraglandular cutaneous, pulmonary, gastrointestinal, cardiac, neurological, and renal symptoms may occur in SS patients [13,14,15,16,17]. Keratoconjunctivitis associated with dry eyes is a severe, extremely uncomfortable symptom of this disease. In this study, injury of the corneal epithelium and decreased numbers of conjunctival goblet cells resembling keratoconjunctivitis with SS patients was observed in the SS model mice. We previously reported effective treatments of autoimmune lacrimal gland lesions in the SS model mice using eye drops containing immunosuppressive and mucosal-protective agents [11]. The results from this study about ocular lesions in the SS model mice are useful for understanding their therapeutic effects on dry eye as well as the pathogenesis of ocular lesions in SS patients.

Animal models provide useful insights into understanding the pathogenesis and mechanisms of human autoimmune diseases. A variety of animal SS models have been reported [5]. The New Zealand black/New Zealand white (NZB/NZW)F1 mouse strain was one of the first models of spontaneous SS [18]. Animals naturally prone to autoimmune disease have been used in many experimental models, including non-obese diabetes (NOD) mice for autoimmune diabetes (type-I diabetes), and MRL/*lpr* mice bearing *Fas* gene mutations for rheumatoid arthritis (RA) and systemic lupus erythematosus [19,20]. In addition, C57BL6.NOD-*Aec1Aec2* mice carrying two genetic sequences derived from NOD mice and the alymphoplasia (*aly/aly*) mouse strain bearing a mutation of the NF-κB inducing kinase (*NIK*) gene have both been used as SS models [21,22,23]. Understanding the mechanisms of the onset or development of SS-like lesions in these models, including the impairment of T cell differentiation, the dysfunction of T cells, and the imbalance of cytokine production may lead to a better understanding of the human disease [5,19,20,21,22,23].

There are several well-known drug-induced and surgically induced SS models, including a multiorgan autoimmune model with SS-like lesions formed by injection of a neutralizing antibody against CD25 and deletion of regulatory T cells in the peripheral circulation [24]. Neonatal thymectomy of NFS/*sld* mice produced the primary SS model used in this study. Using this model, we previously reported a salivary gland-specific SS autoantigen, described an antigen-specific response of T-helper 1 (Th1) cells, and evaluated several molecular mechanisms and agents for SS therapy [6,7,10,25].

Furthermore, a number of genetically manipulated gene knockout (KO) and transgenic (TG) mouse models have been used to investigate the molecular pathogenesis of SS [5]. *TGF-β1 Receptor* KO, *IL-10* TG, and *IL-14α* TG mice have been reported as spontaneous SS models, and SS-like lesions have been observed in autoimmune regulator (*AIRE*) KO mice [26,27,28,29]. AIRE is a transcription factor important for the autoantigen expression in the thymus [30]. We have also evaluated C-C chemokine receptor (*CCR7)* KO, *RelB* KO, and retinoblastoma-associated protein (*RbAp*) *48* TG mice as SS models [31,32,33]. SS-like autoimmune lesions in the *CCR7* KO and *RelB* KO mice occur through the impairment of T cell differentiation or selection in the thymus [31,32]. RbAp48 in salivary and lacrimal glands is upregulated by estrogen deficiency at the postmenopausal stage in women, and it induces apoptosis of salivary and lacrimal gland cells [33]. SS-like autoimmune lesions in the *RbAp48* TG mice are caused by apoptosis of target cells and a breakdown of local tolerance through abnormal expression of cytokines in target organs [33].

Focusing on the ocular lesions in SS, *AIRE* KO and C57BL6.NOD-*Aec1Aec2* mice are useful for investigating corneal and conjunctival epithelial injury associated with dry eye, since these lesions are more severe than those observed in other SS models [21,22,34,35]. However, the incidence of autoimmune lesions in many SS models is relatively low and the onset is age-dependent. The incidence of SS lesions in the model used in this study was almost 100%, and the lesions occurred in relatively young female mice at 6–8 weeks of age. The ocular lesions observed in this study involved corneal and conjunctival tissue, intraorbital and extraorbital lacrimal glands, and Harderian glands. The model is thus useful for understanding the pathogenesis of the ocular lesions in SS. However, SS is a multifactorial disease, and approaches from diversified standpoints using various models are necessary for defining the precise mechanism of SS.

Tissues present in the orbit include the eyeball, fat, muscle, connective tissue, and lacrimal glands. Extraorbital lacrimal gland tissues have been evaluated in most SS animal model studies, since systemic exocrine glands are targeted in SS, in addition to intraorbital lacrimal glands. The minor salivary glands of the lip, oral palate, and oral mucosa and major salivary glands including the submandibular, sublingual, and parotid glands, are targeted in SS patients. Harderian glands and intraorbital lacrimal glands are present in mice, but not in humans, and act as accessories to the lacrimal glands by secreting mucus or lipids. Accessory lacrimal glands are present in the conjunctival tissue of humans.

Tear VEGF levels were significantly lower in the SS model mice than in control mice. VEGF is a potent angiogenic factor and is important for tissue regeneration [36]. Decreased tear VEGF levels may be associated with inflammation and tissue injury of ocular tissues in dry eye syndrome. Therapeutic agents targeting VEGF and its receptor have been described in patients with dry eye [37]. MIP-1α/CCL3 levels were higher in the tears of the SS model mice than in the tears of control mice. It has been reported that the MIP-1α level is increased in the saliva of patients with primary SS compared with healthy controls [38], and the findings in this SS mouse model also suggest that MIP-1α plays a role in the inflammation of the ocular lesions.

TGF-β is a pleiotrophic cytokine with either proinflammatory or anti-inflammatory activity [39], and it is upregulated in the corneal tissues of SS patients [40]. In this study, TGF-β mRNA expression was significantly higher in the SS model mice than in control mice, which is consistent with the finding of dry eye-induced keratoconjunctivitis in the SS model mice. IL-6 and TNF-α upregulation in the corneal tissues of the SS model mice is also consistent with the presence of inflammatory lesions of corneal tissue. CXCL12 plays key roles in the development of corneal epithelium, function of corneal fibroblasts, and angiogenesis of corneal tissues [41,42,43]. CXCL12 may also contribute to inflammation or regeneration of corneal lesion in SS pathogenesis.

As in previous studies of the salivary and lacrimal glands in SS patients and animal models [1,4,5], CD4^+^ T cells were the predominant infiltrating immune cells in the lacrimal glands of this SS model at 10 weeks of age. The subsets of immune cells found in autoimmune lesions are age-dependent. In this SS model, the B-cell population of autoimmune lesions increases with age. For cytokine expression of the extraorbital lacrimal glands in SS models, the finding of TNF-α and TGF-β mRNA upregulation in the inflammatory lesions in this mouse SS model is consistent with reports of the upregulation of TNF-α in the salivary glands of SS patients [44,45].

In summary, tear-content analysis as well as histological, immunological, and gene expression of corneal and conjunctival tissue and inflammation of the lacrimal glands and accessory glands in this SS model adds to the understanding of the pathogenesis of dry eye-induced ocular lesions. It is also useful for developing novel clinical diagnostic and therapeutic tools for managing SS.

## 4. Materials and Methods

### 4.1. Mice

Female NFS/N mice with mutant *sld* were reared in a specific pathogen-free mouse colony and a thymectomy was performed on day 3 of life to produce the SS model. The thymectomy on day 3 after birth was conducted under anesthesia by a suction technique using an aspirator. At 4 weeks after the thymectomy, peripheral blood monocytes from the tail veil were analyzed by flow cytometry. Completeness of thymectomy was confirmed by evaluating numbers of Thy1.2^+^ T cells (less than 7%). The study was conducted following the Fundamental Guidelines for Proper Conduct of Animal Experiments and Related Activities in Academic Research Institutions under the jurisdiction of the Ministry of Education, Culture, Sports, Science and Technology of Japan. The protocol was approved by the Committee on Animal Experiments of Tokushima University and Biological Safety Research Center (Permit Number: T27-7, 14 April 2015). All experiments were performed under anesthesia, and all efforts were made to minimize suffering. Mice at 10–12 weeks old of age were used in this study.

### 4.2. Tear Secretion Measurements

The tear secretion volume was determined using a previously described method [11]. In brief, to measure tear secretion in pilocarpine-stimulated mice, mice were anesthetized with ketamine (60 mg/kg body weight) and xylazine (6 mg/kg), and then intraperitoneally injected with pilocarpine (2.5 mg/kg, Wako, Osaka, Japan). Tear volume was determined by measuring the length of the phenol-red thread (Showa Yakuhin Kako Co., Ltd., Tokyo, Japan) in contact with the eye at 5 min and 10 min after pilocarpine injection and reported as tear volume/body weight.

### 4.3. Cytokines, Chemokines, and Growth Factors in Tear

Twenty factors were quantified simultaneously using a Cytokine 20-Plex Mouse Panel Luminex assay kit (Invitrogen Corporation, Carlsbad, CA, USA) following the manufacturer’s instructions. The levels of cytokines, chemokines, and growth factors present in tears, including GM-CSF, IFN-γ, IL-1α, IL-1β, IL-2, IL-4, IL-5, IL-6, IL-10, IL-12 (p40/p70), IL-13, IL-17, TNF-α, CXCL10, CXCL1, MCP-1, CXCL9, MIP-1α, FGF, and VEGF, were determined using the Multiplex Bead Immunoassay. In brief, magnetic 20-plex antibody beads suspension (25 μL per well) was added to a microplate. Following washing with wash solution with magnetic bead washing equipment ELx405 (BioTek, Instrumentes, Inc., Winooski, VT, USA), 50 μL of incubation buffer and 50 μL of standards/samples diluted in assay diluent solution were added to the wells. Incubation was performed overnight at 4 °C, protected from light with continuous shaking. Following washing, 100 μL of the biotinylated detection antibody solution was added and incubated for 1 h at room temperature on an orbital shaker. After three washing steps, 100 μL of streptavidin-RPE dilution was added and incubated at room temperature on the shaker. After 30 min of incubation and washing, the plate was subsequently analyzed with Luminex200 system (Merk-Millipore Corporation, Billerica, MA, USA) and quantified using Milliplex^TM^ Anlalyst (Merk-Millipore Corporation).

### 4.4. Evaluation of Ocular Surfaces

A 1–2-μL volume of 2% fluorescein solution (Fluorescite^®^ intravenous injection 500 mg; Alcon^®^, Tokyo, Japan) in physiological saline was applied to the cornea by micropipette, and the residual liquid around the eye was washed with saline and gently wiped with filter paper. After fluorescein staining, the cornea was examined for corneal epithelial disorders using a slit-lamp microscope with a blue filter which was evaluated using a previously described method with modifications [11].

### 4.5. Histological Evaluations

After the mice were sacrificed, all of the organs were removed and fixed in 10% phosphate-buffered formaldehyde (pH 7.2). Formalin-fixed tissue sections containing lacrimal glands were stained with hematoxylin eosin (H&E). Corneal and conjunctival tissues were stained with periodic acid-Schiff (PAS).

### 4.6. Immunofluorescence Staining

Frozen sections of lacrimal gland tissues were fixed in 4% paraformaldehyde in phosphate buffer, blocked with Blocking One Histo (Nacalai Tesque, Kyoto, Japan), and then stained with Alexa 546-conjugated rat anti-mouse CD4, CD8, CD19, or F4/80 monoclonal antibodies (eBiosciences, San Diego, CA, USA) overnight. After washing three times with PBS, nuclear DNA was stained with 4′,6-diamdino-2-phenylindole dihydrochloride (DAPI, Invitrogen, Carlsbad, CA, USA). Sections were observed with a PASCAL confocal laser-scanning microscope (LSM: Carl Zeiss, Jena, Germany) at 400× magnification. The LSM image browser version 3.5 (Carl Zeiss, Oberkochen, Germany) was used for image acquisition.

### 4.7. Flow Cytometry Analysis

Lymphocytes infiltrating into the lacrimal glands were isolated by dispersing the tissues with 1 mg/mL collagenase component solution (Wako), followed by density gradient centrifugation using Histopaque-1083 (Sigma-Aldrich, St. Louis, MO, USA). Lymphocytes from lacrimal gland tissues were stained with antibodies against PE-Cy7-conjugated anti-mouse CD4 mAb (TONBO Biosciences, San Diego, CA, USA), fluorescein isothiocyanate (FITC)-conjugated anti-mouse CD8 mAb (eBioscience, San Diego, CA, USA), PE-Cy5.5-conjugated anti-mouse CD19 mAb (TONBO Biosciences), and APC-Cy7-conjugated anti-mouse F4/80 mAb (BioLegend, San Diego, CA, USA). A FACScant flow cytometer (BD Biosciences, San Jose, CA, USA) was used to identify the cell populations based upon the surface expression profile. Data were analyzed using FlowJo FACS Analysis software (Tree Star Inc., Ashland, VA, USA).

### 4.8. Quantitative Real-Time Reverse Transcription-Polymerase Chain Reaction (RT-PCR)

Total RNA was extracted from lacrimal gland tissues and corneas using ISOGEN (Wako Pure Chemical Industries, Ltd., Osaka, Japan) and was reverse transcribed. Transcriptions of target genes and β-actin were prepared using a 7300 Real-Time PCR System (Applied Biosystems, Foster City, CA, USA) with SYBR Premix Ex Taq (Takara Bio, Shiga, Japan). The primer sequences used were as follows: 

TGF-β, 5′-GACCGCAACAACGCCATCTAT-3′ (forward) and 5′-GGCGTATCAGTGGGGGTCAG-3′ (reverse); TNF-α, 5′-ATGAGAAGTTCCCAAATGGC-3′ (forward) and 5′-CTCCACTTGGTGGTTTGCTA-3′ (reverse); IL-6, 5′-TCCTTCCTACCCCAATTTCC-3′ (forward) and 5′-GCCACTCCTTCTGTGACTC-3′ (reverse); CXCL12, 5′-CTTCATCCCCATTCTCTCA-3′ (forward) and 5′-GACTCTGCTCTGGTGGAAGG-3′ (reverse); CXCL10, 5′-CCTTCACCATGTGCCACGC-3′ (forward) and 5′-TCTTACATCTGAAATAAAAGAGCTCAGGT-3′ (reverse); MCP-1, 5′-CTGGATCGGAACCAAATGAG-3′ (forward) and 5′-TGAGGTGGTTGTGGAAAAGG-3′ (reverse); TSLP, 5′-CAGCTTGTCTCCTGAAAATCG-3′ (forward) and 5′-AAATGTTTTGTCGGGGAGTG-3′ (reverse); CX3CL1, 5′-TGAGAGTGAGGAAGCCAACC-3′ (forward) and 5′-GGAACCAACAAAGTCCGATG-3′ (reverse); IL-18, 5′-GTGACCCTCTCTGTGAAGGATA-3′ (forward) and 5′-TGTGTCCTGGAACACGTTTC-3′ (reverse); β-actin, 5′-GTGGGCCGCTCTAGGCACCA-3′ (forward) and 5′-CGGTTGGCCTTAGGGTTCAGGGGG-3′ (reverse).

Relative mRNA expression of each transcript was normalized against β-actin mRNA.

### 4.9. Statistical Analysis

Treatment group means were compared by an unpaired Student’s *t*-test. A probability (*p*) value < 0.05 was defined as statistically significant.

## 5. Conclusions

Analyzing the ocular lesions in SS models adds to the understanding of the complicated pathogenesis and aids in the development of new SS therapies.

## Abbreviations

SS	Sjögren’s syndrome
VEGF	Vascular endothelial growth factor
MIP-1α	Macrophage inflammatory protein-1α
TNF-α	Tumor necrosis factor-α
TGF-β	Transforming growth factor-β
CXCL9	C-X-C motif ligand 9
CXCL12	C-X-C motif ligand 12
